# Chaos Fusion Mutation-Based Weighted Mean of Vectors Algorithm for Linear Antenna Array Optimization

**DOI:** 10.3390/s25206482

**Published:** 2025-10-20

**Authors:** Zhuo Chen, Yan Liu, Liang Dong, Anyong Liu, Yibo Wang

**Affiliations:** 1 School of Physics and Electronic Information, Yunnan Normal University, Kunming 650504, China; cz15656171360@163.com (Z.C.); 17587074972@163.com (A.L.); 17387599197@163.com (Y.W.); 2 Yunnan Province China-Malaysia HF-VHF Advance Radio, Astronomy Technology International Joint Laboratory, Yunnan Observatory, Chinese Academy of Sciences, Kunming 650011, China; dongliang@ynao.ac.cn

**Keywords:** linear antenna arrays, pattern synthesis, sidelobe suppression, null steering, metaheuristic optimization

## Abstract

**Highlights:**

**What are the main findings?**

- CFMINFO is a weighted-mean optimizer with good-lattice initialization, STC chaos, and cloud mutation, designed for constrained array synthesis.
- It optimizes both element spacings and amplitudes, ensuring prescribed deep-null steering.

**What is the implication of the main finding?**

- CFMINFO achieves SLL ≈ −32.30 dB and a −125.1 dB deep null at 104°, while preserving the main lobe for effective interference suppression.
- It outperforms PSO/GA/IWO/HSA/FPA, demonstrating the best Friedman rank ≈ 1.36 on 7 CEC2020 constrained optimization tasks.

**Abstract:**

This study proposes the Chaos Fusion Mutation-Based Weighted Mean of Vectors Algorithm, an advanced optimization technique within the weighted mean of vectors (INFO) framework for synthesizing unequally spaced linear arrays. The proposed algorithm incorporates three complementary mechanisms: a good-point-set initialization to enhance early population coverage, a sine–tent–cosine (STC) chaos–based adaptive parameterization to balance exploration and exploitation, and a normal-cloud mutation to preserve diversity and prevent premature convergence. Array-factor (AF) optimization is posed as a constrained problem, simultaneously minimizing sidelobe level (SLL) and achieving deep-null steering, with penalties applied to enforce geometric and engineering constraints. Across diverse array-synthesis tasks, the proposed algorithm consistently attains lower peak SLLs and more accurate nulls, with faster and more stable convergence than benchmark metaheuristics. Across five simulation scenarios, it demonstrates robust superiority, notably surpassing an enhanced IWO in the combined objectives of deep-null suppression and maximum SLL reduction. In a representative engineering example, we obtain an SLL and a deep null of approximately −32.30 and −125.1 dB, respectively, at 104°. Evaluation of the CEC2020 real-world constrained problems confirms robust convergence and competitive statistical ranking. For reproducibility, all data and code are publicly accessible, as detailed in the Data Availability section.

## 1. Introduction

The primary function of antenna arrays is to enhance directionality, increase gain coefficients, and achieve desired directional characteristics. However, achieving a narrow first null beamwidth (FNBW) while suppressing the sidelobe level (SLL) presents a design trade-off, as reducing SLL often comes at the expense of gain and beamwidth. Resultantly, in practical design, minimizing SLL without limiting the FNBW has become a critical criterion for optimizing antenna array performance. This balance is essential for improving the overall performance of antenna arrays in communication and astronomical observations.

Millimeter-wave (mmWave) communication has become a key enabler for emerging 5G/6G applications, such as intelligent transportation systems, vehicular networks, and urban sensing. In such scenarios, accurate channel parameter estimation is crucial for achieving reliable connectivity under dynamic traffic conditions [1]. High-performance antenna arrays play a central role in this process, as their ability to suppress sidelobes and generate deep nulls directly impacts the robustness of beamforming and channel estimation. These requirements motivate the constrained array–synthesis framework studied in this paper, where sidelobe level minimization and deep–null placement are explicitly enforced.

In the 1960s, Unz introduced the concept of “arbitrarily distributed elements” [2], which marked the advent of sparse arrays and stimulated research on non-uniform array designs to suppress grating lobes and achieve low sidelobe performance. Subsequent studies proposed numerous optimization methods. Harrington applied the Fourier series expansion of array differences to propose a perturbation theory for sidelobe reduction [3], while Andreasen examined the relationship between SLLs and element count [4]. These studies elucidated the effect of aperture size on main lobe width and laid a strong theoretical foundation for sparse array research.

In electromagnetic field problems and antenna optimization, nature-inspired population-based algorithms dominate stochastic optimization techniques. John Holland pioneered the Genetic Algorithm (GA) [5] in the 1960s, later refining it with colleagues at the University of Michigan (1960s–1970s), introducing the concept of natural adaptation into computation.

Subsequent years witnessed the emergence of numerous metaheuristic algorithms, including Differential Evolution (DE) [6], Particle Swarm Optimization (PSO) [7], Ant Colony Optimization (ACO) [8], Firefly Algorithm (FA) [9], Cuckoo Optimization Algorithm (COA) [10], Harmony Search (HS) [11], Backtracking Search Algorithm (BSA) [12], and Grey Wolf Optimization (GWO) [13]. Recent studies have focused on optimizing non-uniform and unequally spaced arrays, including linear and spiral configurations, to minimize SLLs and enforce deep–null constraints. For example, an enhanced Harris Hawks Optimization (EHHO) algorithm has recently been proposed for non-uniform spiral array design, effectively reducing peak SLLs (PSLLs) and suppressing grating lobes [14].

This study refines the INFO [15] by incorporating Sine–Tent–Cosine chaotic mapping, good lattice point initialization, and multi-normal cloud mutation strategies, yielding the CFMINFO algorithm. CFMINFO optimizes amplitude and spacing uniformity in linear arrays, achieving lower SLLs and enhancing directivity. It also optimizes antenna patterns for deep nulls at specified angles, meeting noise suppression requirements.

In the following work, Section 2 presents a constrained linear array–synthesis formulation that minimizes the maximum sidelobe level (SLL) while simultaneously satisfying FNBW and deep-null constraints. Section 3 introduces three complementary mechanisms—(i) a Hua-style good-lattice initializer to enhance early population coverage; (ii) a sine–tent–cosine (STC) chaotic schedule to balance exploration and exploitation throughout the run; and (iii) a normal-cloud mutation to maintain diversity near convergence—thereby yielding CFMINFO, a lightweight and robust enhancement of INFO. Under the same constraint set and computational budget as in Section 2, the proposed method attains a maximum SLL of −32.30 dB and a deep null of −125.1 dB at 104°; it outperforms PSO/GA/IWO/HSA/FPA, with comparative and ablation results detailed in Section 4, and conclusions and future work provided in Section 5.

Contributions. This paper makes the following four contributions:

- A Hua-style good-lattice initializer that improves early population coverage;
- A sine–tent–cosine (STC) chaotic schedule that balances exploration and exploitation;
- A normal-cloud mutation that maintains diversity near convergence;
- A constrained array-synthesis setup that achieves −32.30 dB max-SLL and a −125.1 dB deep null at 104°, outperforming PSO/GA/IWO/HSA/FPA under identical budgets.

## 2. Problem Formulation

We adopt the standard definitions of the linear-array factor and radiation pattern from classical antenna texts [16,17,18]. For completeness, the working formulas used in this study are restated here, with the focus placed on the constrained optimization setup.

In this manuscript, we consider the symmetric linear array antenna (SLAA), where 2N isotropic elements are arranged symmetrically about the array center. The symmetry simplifies the mathematical formulation while allowing unequal inter-element spacings and/or non-uniform excitations to be treated as decision variables in the constrained synthesis.

**Figure 1:**
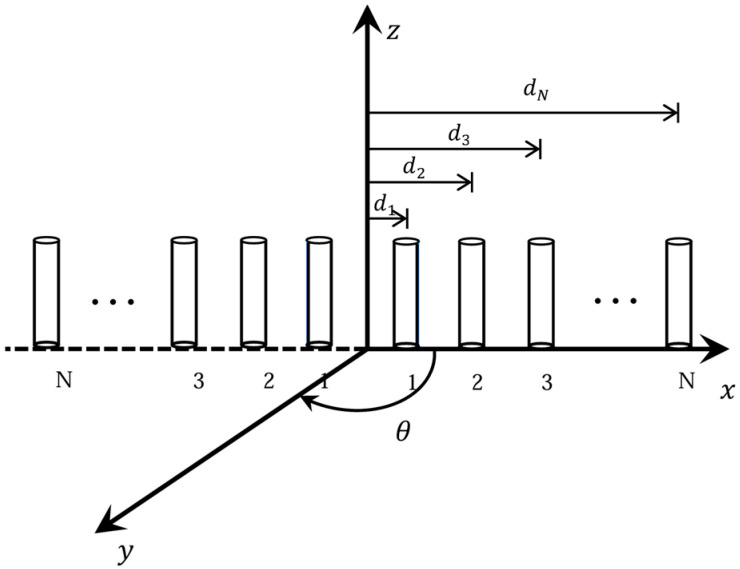
The spatial arrangement of a 2N-element linear array.

For an SLAA composed of 2N isotropic elements, the array factor (AF) can be expressed as follows:

(1) $$AF(\theta )=2\sum_{n-1}^{N}A_{n}\cos(\alpha_{n}+\beta_{n}+kd_{n}\cos\theta )$$

where *A_n_* and $\alpha_{n}$ depicts the excitation amplitude and phase of the n-th element; *d_n_* signifies its distance from the center of the array to the *n*-th element; *θ* indicates the sidelobe scanning angle, and *k* refers to the wavenumber equivalent to 2*π*/*λ*, $\beta_{n}$ is the progressive phase for each element; when beam scanning is not required, set $\beta_{n}=0$. Here, the cosine function is employed to leverage the symmetry of the array. Note that $d_{n}$ represents the spacing difference relative to the center of the array, rather than the absolute spacing. In practical applications, we need to determine the values of *d_n_* for each element according to the specific array configuration. For detailed derivations of the AF forms and constraint definitions, please see Appendix A. Figure 1 is the spatial arrangement of a 2N-element linear array in terms of its geometric position distribution.

Ultimately, the computation of the maximum Sidelobe Level (SLL) on the array pattern can be undertaken,

(2) $$AF_{dB}(\theta )=20\log(\frac{AF(\theta )}{\max(\left|AF(\theta )\right|)}).$$

Due to array symmetry, the radiation pattern is also symmetric, with the main beam oriented along the normal axis (*θ* = 0). In array antenna studies, the radiation pattern is a key indicator of diverse algorithmic performance. Reductions in SLLs and changes in main beam width indicate algorithm effectiveness. The subsequent sections present an optimized linear array configuration using radiation patterns and comparative analyses with alternative algorithms.

## 3. Proposed Method

INFO [15] is an enhanced weighted mean-based optimization method that updates vector positions through three core procedures: initialization, updating rules, vector combining, and local search.

During initialization, the INFO algorithm comprises a population of Np vectors within a D-dimensional search domain that g represents the number of iterations ($X_{l,j}^{g}=\left\{x_{l,1}^{g},x_{l,2}^{g},x_{l,3}^{g},...,x_{l,D}^{g}\right\},l=1,2,3,\ldots ,Np$). At this stage, several control parameters for the INFO algorithm are introduced and defined. Notably, there are two key parameters, the weighted average factor δ and the scaling factor σ. These two parameters do not need to be adjusted by the user and change dynamically based on generation. The INFO algorithm uses a simple method to generate the initial vectors called random generation.

During the updating rules phase, new vectors are produced using the mean rule and convergence acceleration, as expressed in Equations (3) and (4).

(3) $$z1_{l}^{g}=\left\{\begin{array}{c} x_{l}^{g}+\sigma \times MeanRule+randn\times \frac{x_{bs}-x_{a1}^{g}}{\left(f(x_{bs})-f(x_{a1}^{g})+1\right)}\begin{array}{cc} & rand<0.5 \end{array}\\ x_{a}^{g}+\sigma \times MeanRule+randn\times \frac{x_{a2}^{g}-x_{a3}^{g}}{\left(f(x_{a2}^{g})-f(x_{a3}^{g})+1\right)}\begin{array}{cc} & rand>0.5 \end{array} \end{array}\right.$$

(4) $$z2_{l}^{g}=\left\{\begin{array}{c} x_{bs}+\sigma \times MeanRule+randn\times \frac{x_{a1}^{g}-x_{b}^{g}}{\left(f(x_{a1}^{g})-f(x_{a2}^{g})+1\right)}\begin{array}{cc} & rand<0.5 \end{array}\\ x_{bt}+\sigma \times MeanRule+randn\times \frac{x_{a1}^{g}-x_{a2}^{g}}{\left(f(x_{a1}^{g})-f(x_{a2}^{g})+1\right)}\begin{array}{cc} & rand>0.5 \end{array} \end{array}\right.$$

wherein, $x_{l}^{g}$, $x_{bs}$, $x_{bt}$ indicate the *l*-th vector, the best and the better solution in the population during the $g^{th}$ generation, and the MeanRule represents the update rule, a1 ≠ a2 ≠ a3 denote three randomly selected integers within the range [1, *Np*]. The vector scaling rate (*σ*) is expressed in Equation (5), while α varies with the exponential function defined in Equation (6):

(5) σ=2α×rand−α

(6) $$\alpha =2\exp(-4\times \frac{g}{\max g})$$

In the vector combination stage, the two previously calculated vectors are merged with the vector $x_{l}^{g}$ to generate the new vector $u_{l}^{g}$ where *rand* < 0.5. The new vector $u_{l}^{g}$, formulated in Equation (7), incorporates two independent random variables: *rand*_1_ and *rand*_2_.

(7) $$u_{l}^{g}=\left\{\begin{array}{c} z1_{l}^{g}+\mu \cdot |z1_{l}^{g}-z2_{l}^{g}|\;if\;rand_{1}<0.5,\;rand_{2}<0.5\\ z2_{l}^{g}+\mu \cdot |z1_{l}^{g}-z2_{l}^{g}|\;if\;rand_{1}<0.5,\;rand_{2}>0.5\\ x_{l}^{g}\;if\;rand_{1}>0.5,\;rand_{2}>0.5 \end{array}\right.$$

The local search stage update $u_{l}^{g}$ using newly defined rules and random factors to prevent the algorithm from deception and entrapment into locally optimal solutions. Based on two randomly determined conditions, different formulas can be used to update the vector $u_{l}^{g}$.

(8) $$u_{l}^{g}=\left\{\begin{array}{cc} x_{bs}+randn\times (MeanRule+randn\times (x_{bs}^{g}-x_{a1}^{g})) & if\;rand_{1}<0.5,rand_{2}<0.5\\ x_{rnd}+randn\times (MeanRule+randn\times (v_{1}\times x_{bs}-v_{2}\times x_{rnd})) & if\;rand_{1}<0.5,rand_{2}>0.5 \end{array}\right.$$

where $x_{rnd}$ implies the new solution, and $v_{1},v_{2}$ signifies two random numbers.

Unlike conventional population-based optimization algorithms inspired by biological or natural behaviors, the INFO algorithm employs a strictly mathematical framework founded on weighted vector averaging and dynamic feedback. Structurally, it employs a modular three-stage design: (1) a weighted mean-based update mechanism utilizing wavelet-regulated coefficients, (2) a vector combining process that promotes diversity and balance, and (3) a local search phase focused on convergence refinement.

This structure enables INFO to effectively balance exploration and exploitation, while the dynamic adjustment of key parameters (e.g., the scaling factor and convergence rate) mitigates premature convergence and enhances stability.

Compared to mainstream optimization algorithms, INFO exhibits distinct structural differences. While PSO [7] depends on individual and global best memories, DE [6] employs difference-based perturbation, and GA [5] relies on genetic operators, such as crossover and mutation. INFO, in contrast, operates without historical memory or stochastic recombination. Instead, it applies deterministic mathematical rules with random modulation, providing enhanced theoretical and convergence clarity.

This shift from heuristic analogies to mathematical modeling represents a new paradigm in optimization algorithm design, strengthening theoretical and practical efficiency.

The INFO algorithm holds great potential for improvement. This study introduces CFMINFO, which optimizes initial populations through Good Lattice Point Set initialization. It further integrates STC chaotic map parameterization with normal cloud mutation to boost accuracy and avoid local optima. The next three sections detail these strategies.

### 3.1. Initialization Improvement

To mitigate the issue of uneven population distribution during initialization, this study adopts the Good Lattice Point Set initialization concept [19], originally proposed by the Chinese mathematician, Mr. Hua Luogeng.

Let us consider Hs to represent a unit cube positioned within an s-dimensional Euclidean space, wherein there resides a specific point set is given as shown in (9) as follows:

(9) $$P_{n}(k)=\left\{\left(\left\{r_{1}^{n}\times k\right\},\left\{r_{2}^{n}\times k\right\},\left\{r_{3}^{n}\times k\right\},\ldots ,\left\{r_{s}^{n}\times k\right\}\right),1\leq k\leq n\right\}$$

**Figure 2:**
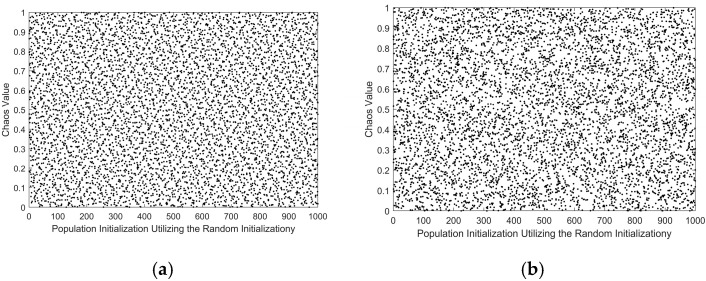
(**a**) the Good Lattice Point Set Map; (**b**) the Random Initialization Population Map.

The latest initialization based on the good lattice point set is presented as shown in Equation (10).

(10) $$x_{i}(k)=(ub_{i}-lb_{i})\left\{P_{n}(k)\right\}+lb_{i}$$

where *i* represents each dimension, *ub* indicates the upper bound and *lb* signifies the lower bound. Figure 2 depicts the population distribution diagram based on the improved initialization.

### 3.2. Parameterization of the STC Composite Chaotic Map

The STC (Sine–Tent–Cosine) map [20] models a complex chaotic behavior in deterministic nonlinear systems, exhibiting sensitivity, aperiodicity, and randomness. As a composite chaotic mapping with state values distributed over (0, 1), it finds broad applicability across domains. In optimization algorithms, integrating the STC map with random perturbations improves convergence speed and characteristics, making it a highly effective optimization mechanism.

The STC chaotic map integrates the Sine chaotic and the Tent chaotic maps, defined in Equations (11) and (12), respectively.

(11) $$x_{i+1}=S(r,x_{i})=r\sin(\pi x_{i})$$

(12) $$x_{i+1}=T(r,x_{i})=\left\{\begin{array}{cc} 2rx_{i} & if\;x_{i}<0.5\\ 2r(1-x_{i}) & if\;x_{i}>0.5 \end{array}\right.$$

Then, the literature [20] proposes a chaotic system based on cosine transformation (CTBCS) for generating combined chaotic maps with complex behaviors.

The final obtained STC chaotic map is shown in Equation (13).

(13) $$x_{i+1}=\left\{\begin{array}{c} \cos(\pi (r\sin(\pi x_{i})+2(1-r)x_{i}+\mu ))\begin{array}{cc} & x_{i}<0.5 \end{array}\\ \cos(\pi (r\sin(\pi x_{i})+2(1-r)(1-x_{i})+\mu ))\begin{array}{cc} & x_{i}>0.5 \end{array} \end{array}\right.$$

### 3.3. Normal Cloud Mutation

The Normal Cloud Mutation Strategy, based on the Normal Cloud Model, enhances population diversity and prevents the algorithm from converging to local optima.

Among various uncertainties, randomness and fuzziness are the most dominant. To address these challenges, the literature [21] introduced the cloud model, which enables the transformation between quantitative data and qualitative concepts. The cloud model is characterized by three mathematical parameters: expected value (Ex), entropy (En), and hyper-entropy (He).

**Figure 3:**
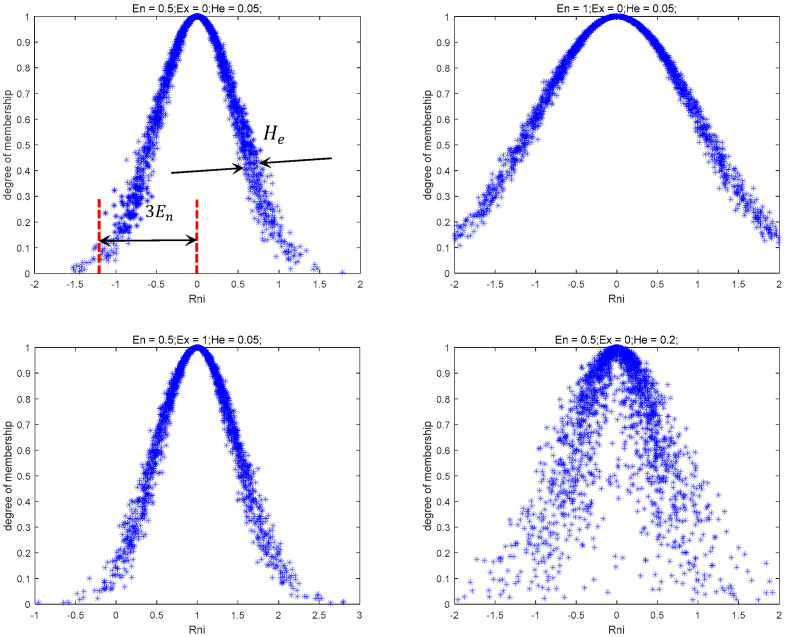
The resulting Normal Cloud Model.

According to cloud model theory [22], the Ex represents the search range center, En denotes search range extent, with broader coverage for larger En, and hyper-entropy (He) indicates dispersion of cloud droplets. Figure 3 illustrates this concept, where *R_n,i_* implies a standard normal deviate for the *i*-th dimension of individual *n*. When introducing the Normal Cloud Model into the INFO algorithm, the Normal Cloud operator exhibits three distinct mathematical properties:

(14) $$\left\{\begin{array}{c} Ex=fit(Best\_X)\\ En=\exp(\frac{g}{Maxg})\\ He=En\times 10^{-\xi } \end{array}\right.$$

wherein, ξ is a positive integer. The probability of an individual being selected is calculated using the following formula:

(15) $$p=\exp[-\frac{2{\left(En^{\prime }\right)}^{2}}{{\left(\mu -Ex\right)}^{2}}]$$

(16) $$En^{\prime }=normrnd(En,He)$$

(17) $$\mu =normrnd(Ex,\left|En^{\prime }\right|)$$

Hence, the introduction of normal cloud mutation serves as the third enhancement strategy in this paper, leveraging the configuration of the expected value, entropy, and hyper-entropy of the normal cloud model to explore the location of solutions.

**Figure 4:**
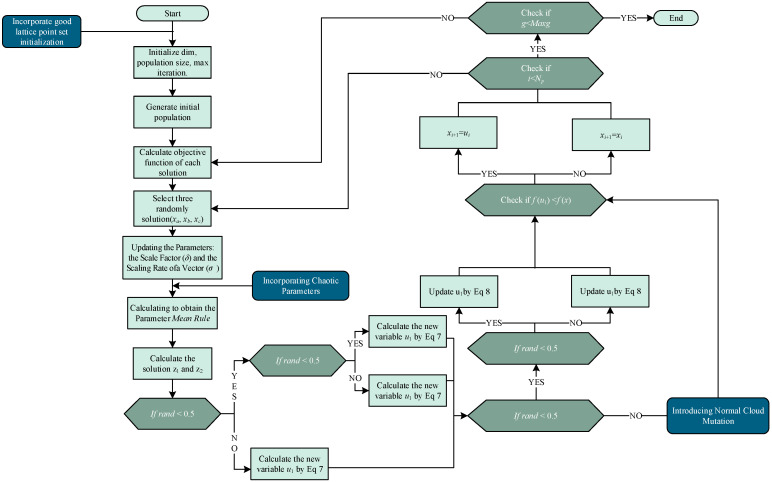
The CFMINFO algorithm flowchart.

CFMINFO that based on the modifications to the initial INFO algorithm according to the above strategies, the final algorithm flowchart is shown in Figure 4.

### 3.4. Comparison of Test Functions

This study employed seven representative RC problems from the CEC2020 real-world constrained optimization benchmark suite [23]: RC15 (Speed Reducer Weight Minimization), RC17 (Tension/Compression Spring Design), RC19 (Welded Beam Design), RC20 (Three-Bar Truss Design), RC23 (Step-Cone Pulley Design), RC28 (Rolling Element Bearing Design), and RC31 (Gear Train Design). These test problems encompass diverse mechanical design applications and constraint complexities, offering a comprehensive platform for algorithmic benchmarking. They exhibit unimodal and multimodal objective landscapes, span low to high dimensions, and include pure inequality and mixed equality/inequality constraints. Such diversity enables a thorough evaluation of optimization methods across varying problem characteristics. Although originally derived from mechanical design, these benchmarks exhibit diverse constraints and multimodal characteristics that closely resemble the challenges of antenna array pattern synthesis, making them well suited for evaluating algorithms developed for antenna array optimization.

This study compares the enhanced CFMINFO algorithm with the original INFO [15] and four established methods: DE [6], GWO [13], SSA [24], and EnMODE [25], the fourth-place finisher in the CEC 2020 Real-World Single-Objective Constrained Optimization Competition. All algorithms were tested on the same benchmark problems with uniform population sizes and maximum iteration counts, each executed over 25 independent runs. Table 1 summarizes the mean and standard deviation (SD) of results across the seven test functions over these 25 trials.

CFMINFO strengthens the original algorithm’s exploration and optimization capabilities, achieving superior performance across seven real-world benchmark problems (Table 1). It effectively balances exploitation and exploration, preventing premature convergence to local optima. The three proposed enhancement strategies ensure a seamless transition to the exploration phase, maintaining a balanced search across global and local scales. Table 2 presents the Friedman test average rankings of the five algorithms.

## 4. Numerical Results

This section presents the simulation results of five examples, each addressing amplitude or position optimization under uniformly spaced and weighted array conditions. For computational simplicity and consistent comparisons, the phase discrepancies among the individual elements are assumed negligible (i.e., $\alpha_{n}$= 0, as defined in Section 2).

### 4.1. Optimization of Element Position Based on FNBW Constraints

**Example 1.** 
*We employ the CFMINFO algorithm to reduce the maximum SLL within the angular range of θ∈ [0°, 78°]∪ [102°, 180°] in a 12-element linear array for maintain the characteristics such as the shape of the main lobe and the beam width, where the excitations of all elements remain uniform (In = 1). Specifically, the positions of the first and last elements are fixed at 0.25λ and 2.75λ ($\frac{2N-1}{x}d$, and d = 0.5λ), respectively. The positions of each element are designated as the optimization variables for the CFMINFO algorithm. The specific definition of the objective function is as follows,*

(18) $$Fitness={\left\{(\max[20\log(\frac{\left|AF(\theta_{i})\right|}{\max(|AF(\theta_{i})|)})]-bb1)/bb1\right\}}^{2}$$

*where* $\theta_{i}\in [0^{\circ },78^{\circ }]\cup [102^{\circ },180^{\circ }]$ *that is the range of sidelobes in a linear array. bb1 represents the ideal maximum SLL that is desired for optimization. In Equation (16), bb1 = −20 dB and *$AF(\theta_{i})$ *calculates the value using Equation (2).*

**Figure 5:**
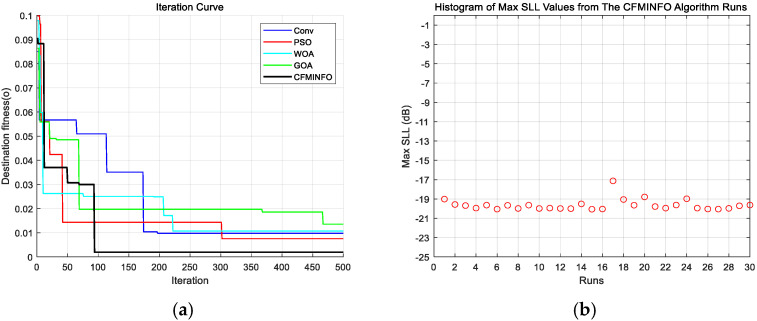
(**a**) Convergence characteristics of CFMINFO and other algorithms for 12-element linear array; (**b**) the distribution of the maximum SLL during 30 runs of CFMINFO.

**Figure 6:**
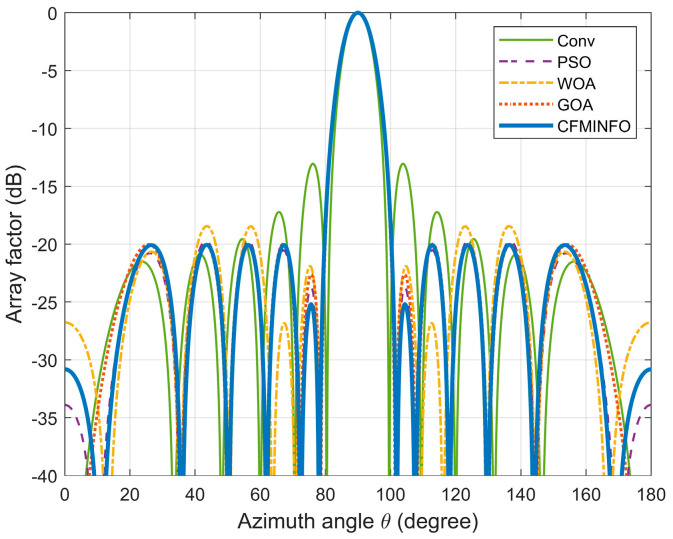
The array pattern of 12-element in Example 1.

**Figure 7:**
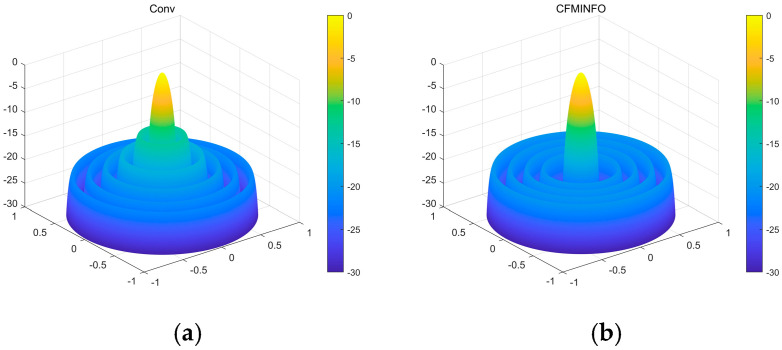
(**a**) The three-dimensional (3D) radiation pattern of an initial 12-element linear array with uniform spacing; (**b**) The three-dimensional (3D) radiation pattern of a 12-element linear array using the CFMINFO algorithm.

In this example, a population of 30 and a maximum of 500 iterations were employed for a six-dimensional solution space. After 30 optimization runs, the convergence behavior of the 12-element linear array using CFMINFO is shown in Figure 5a. Additionally, Figure 5b presents the maximum SLL distribution obtained from CFMINFO. The optimal AF pattern is shown in Figure 6, with Figure 6 and Figure 7 comparing the optimized arrays obtained from PSO [7], the Whale Optimization Algorithm (WOA) [26], and the Grasshopper Optimization Algorithm (GOA) [27]. The 3D radiation pattern resulting from CFMINFO optimization is presented in Figure 7.

Table 3 compares the maximum SLL results of different algorithms, reporting their best, worst, and variance values. The CFMINFO algorithm achieves the lowest SLL of −20.06 dB, outperforming PSO, WOA, and GOA by 0.35, 1.61, and 0.07 dB, respectively. However, this improvement comes at the cost of restricted optimization of the first and last element positions, reducing design flexibility. Across 30 runs, CFMINFO maintains a low SD (0.5891), demonstrating its superior stability and consistency. The detailed element positions are listed in Table 4.

**Example 2.**  *Illustrates the application of CFMINFO in minimizing the SLL within a more intricate 32-element linear array configuration. In this context, CFMINFO treats the position of each array element as an optimization variable. This arrangement facilitates precise control over minimizing the maximum SLL within the angular range of θ∈ [0°, 86°]∪ [94°, 180°]. The excitation amplitudes are uniform, with In = 1 for all array elements. The objective function is Equation (18), where* $\theta_{i}\in [0^{\circ },86^{\circ }]\cup [94^{\circ },180^{\circ }]$ *. In Equation (18), bb1 = −24 dB.*

**Figure 8:**
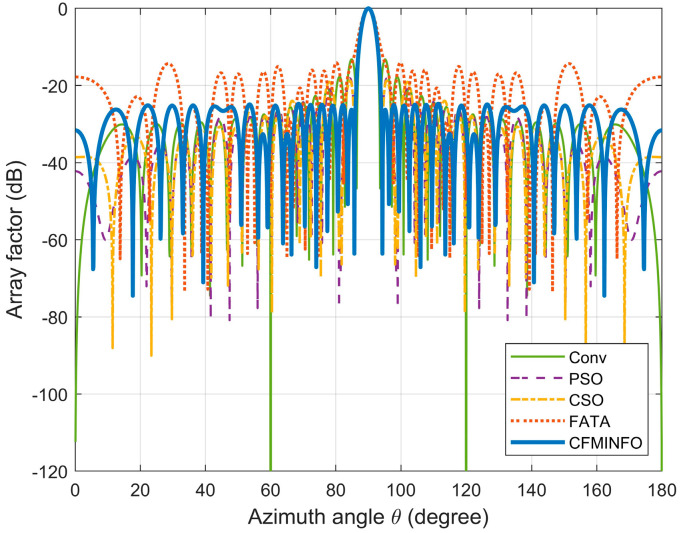
The array pattern of 32-element in Example 2.

In this case study, the CFMINFO algorithm was applied to optimize the element positions of a 32-element linear array, with the objective of achieving the lowest possible maximum SLL, aiming to minimize the maximum SLL while preserving the mainlobe width. Conditions largely mirrored those in Example 1, except that the solution space was set to 16. After 30 rounds of optimization iterations, the optimal AF pattern was obtained (Figure 8) and compared with the results obtained using the optimized Cat Swarm Optimization (CSO) [28], PSO [7], and Fata Morgana Algorithm (FATA) [29].

**Figure 9:**
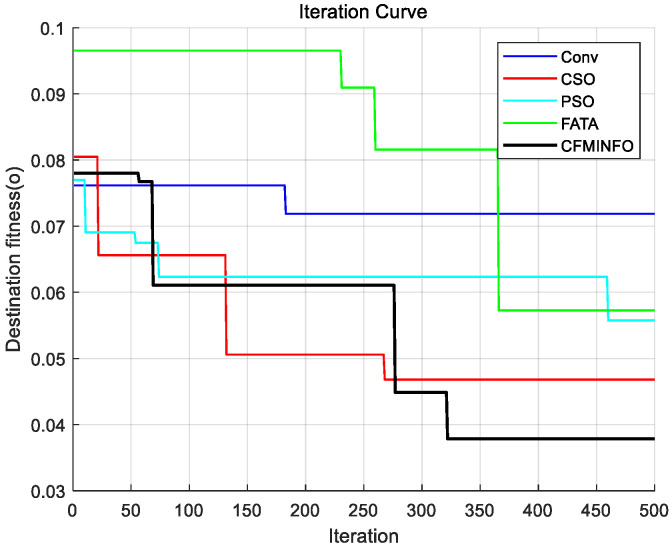
Comparative analysis of iteration curves between CFMINFO and various other algorithms in example 2.

**Figure 10:**
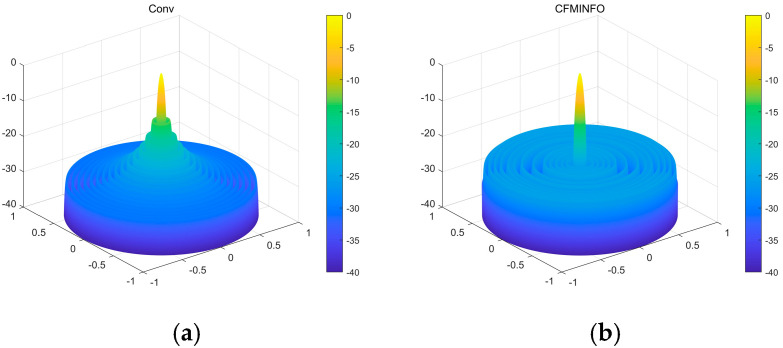
(**a**) The three-dimensional (3D) radiation pattern of an initial 32-element linear array with uniform spacing; (**b**) The three-dimensional (3D) radiation pattern of a 32-element linear array using the CFMINFO algorithm.

Table 3 compares algorithm performance in optimizing maximum SLL and element positions in linear arrays. CFMINFO attained a maximum SLL of −24.74 dB, outperforming FATA, PSO, CSO, and conventional arrays by 10.64, 6.14, 6.54, and 11.24 dB, respectively, demonstrating its superior SLL suppression. CFMINFO outperforms three comparative algorithms in complex scenarios (Figure 9). Figure 10 presents 3D radiation patterns of traditional and CFMINFO-optimized arrays, with element positions in Table 4.

### 4.2. Optimization of Element Amplitude Based on FNBW Constraints

**Example 3.** 
*Optimization focuses on minimizing the maximum SLL of a 10-element linear array (element spacing: 0.5λ) by adjusting excitation amplitudes using CFMINFO. The optimization variables are the excitation amplitudes, constrained to [0, 1], aiming to minimize the maximum SLL within θ* *∈ [0* *°, 76* *°]* *∪ [104* *°, 180* *°]. The objective function is Equation (18), where* $\theta_{i}\in [0^{\circ },76^{\circ }]\cup [104^{\circ },180^{\circ }]$ *, in Equation (18), bb1 = −28 dB.*

**Figure 11:**
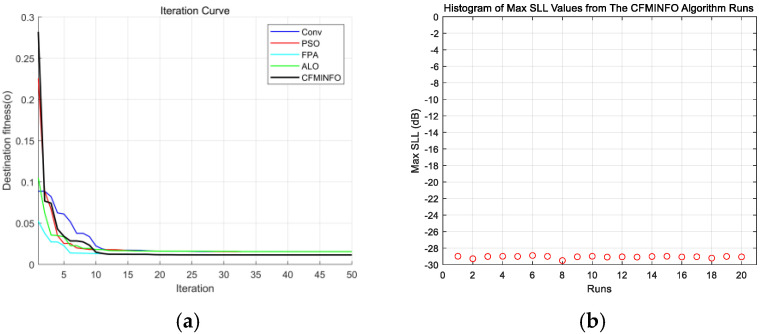
(**a**) Comparative analysis of iteration curves by algorithms in example 3; (**b**) distribution plot of maximum SLL (SLL) for a 10-element array after 20 iterations of CMFINFO optimization in Example 3.

**Figure 12:**
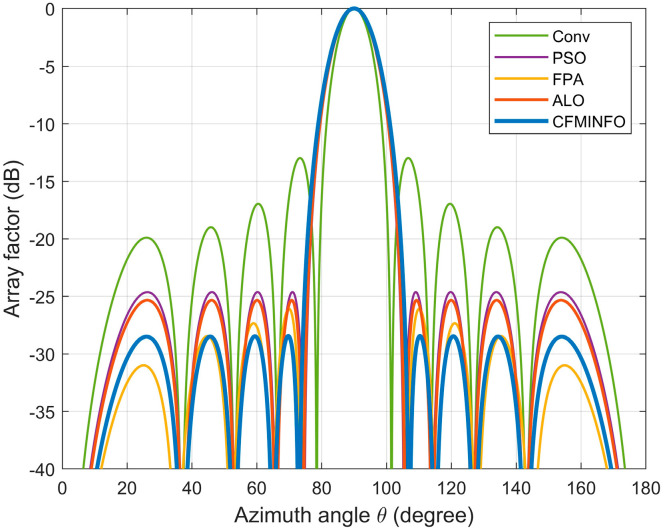
The array pattern of 10-element in Example 3.

**Figure 13:**
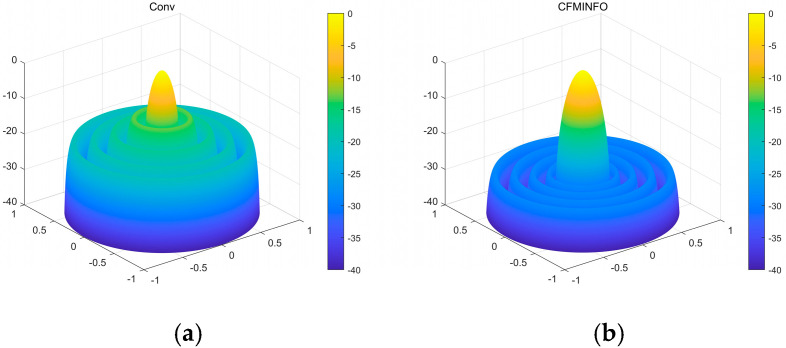
(**a**) the 3D radiation patterns of the traditional 10-element linear array; (**b**) the 3D radiation patterns of the CFMINFO-optimized 10-element linear array.

CFMINFO optimizes the excitation amplitudes of a 10-element linear array to minimize the maximum SLL within [0°, 76°] and [104°, 180°]. Using a 5D solution space with 30 individuals over 500 iterations, Figure 11a compares convergence across 20 runs with other algorithms. Figure 11b illustrates the maximum SLL distribution across 20 CFMINFO runs, and Figure 12 presents the optimal AF compared with PSO [7], FPA [30], and ALO [31]. Table 5 lists the optimized maximum SLL and excitation amplitudes, while Figure 13 compares the 3D radiation patterns of the traditional and CFMINFO-optimized arrays in Example 3.

The results show CFMINFO attains a maximum SLL of −28.44 dB, outperforming the conventional method, PSO, FPA, and ALO by 15.54, 3.82, 3.11, and 2.36 dB, respectively. Table 6 presents the results of 20 runs for the 10-element array, comparing CFMINFO with the other three alternative algorithms. CFMINFO achieves the best, worst, and average SLL with a small SD of 0.5589, demonstrating lower maximum SLL, strong stability, and superior optimization capability.

**Example 4.** 
*The minimization of the maximum SLL for a 16-element linear array is achieved through the application of the CFMINFO algorithm. All settings remain identical to those in Example 3, with the sole exception that the angular range θ of interest now spans [0°, 81°]∪ [99°, 180°]. The objective function is formulated as follows in Equation (18), where *$\theta_{i}\in [0^{\circ },81^{\circ }]\cup [99^{\circ },180^{\circ }]$ *and in Equation (18), bb1 = −29 dB.*

**Figure 14:**
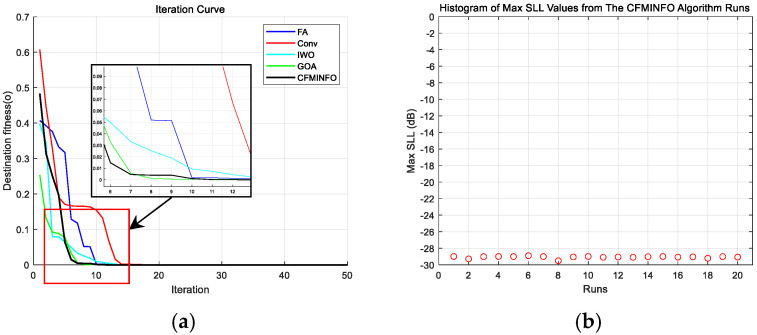
(**a**) the convergence characteristics of CFMINFO for 16-element; (**b**) the distribution of maximum SLLs obtained from 20 runs.

**Figure 15:**
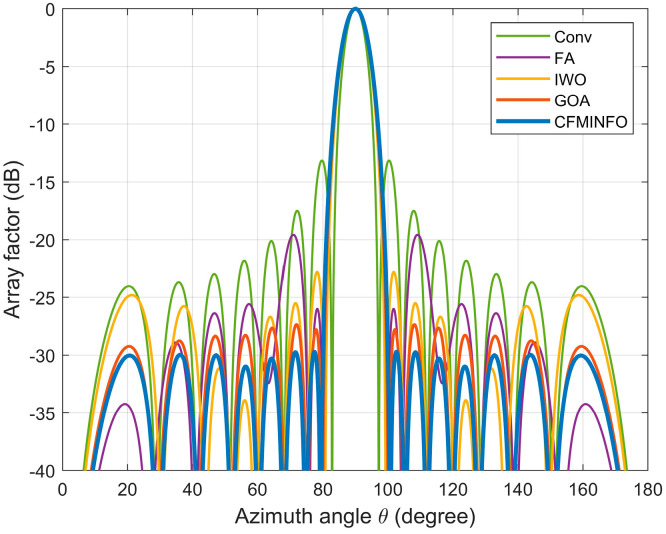
The array pattern of 16-element in Example 4.

To minimize the maximum SLL within the angular range [0°, 81°] ∪ [99°, 180°], the CFMINFO algorithm is applied to optimize the excitation amplitudes of a 16-element linear array. After 20 optimization runs, Figure 14a illustrates the convergence characteristics of CFMINFO for the 16-element linear array, while Figure 14b presents the maximum SLL distribution from 20 runs. Figure 15 compares the optimal AF obtained by CFMINFO, IWO [32], FA [9], and GOA [27]. Table 5 presents optimized maximum SLLs and excitation amplitudes. CFMINFO attains −28.10 dB, outperforming FA, CS, CABMO, and IWO by 3.83, 3.09, 2.23, and 1.71 dB, respectively.

**Figure 16:**
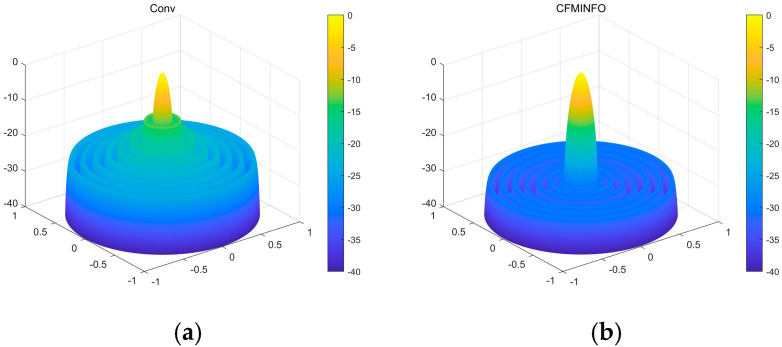
(**a**) the 3D radiation patterns of the traditional 16-element linear array; (**b**) the 3D radiation patterns of the CFMINFO-optimized 16-element linear array.

Table 6 presents the performance of CFMINFO versus other algorithms over 20 runs for the 16-element linear array. CFMINFO consistently outperforms in best, worst, and average SLLs, exhibiting a low SD of 0.1330 relative to the FA. Figure 16 compares the 3D radiation patterns of the conventional 16-element linear array and the CFMINFO-optimized array. In this case, the CFMINFO algorithm attains a lower maximum SLL with good stability, demonstrating superior optimization performance over other algorithms.

### 4.3. Optimization of Element Amplitude Based on FNBW Constraints and Lower Nulling Depth

**Example 5.** 
*CFMINFO is utilized to minimize the SLL while establishing a particular lower nulling depth. A single null is intentionally positioned at a direction of 104°. The other parameters for this optimization remain consistent with those employed in previous examples, with the sole exception of the algorithm being run for 1000 iterations. The optimal excitation amplitudes obtained after the optimization process are tabulated in Table 7. The objective function is formulated as follows:*

(19) $$Fitness=\left[{\left(\left(\max[20\log(\frac{\left|AF(\theta_{i})\right|}{\max(AF(\theta_{i}))})]-bb1\right)/bb1\right)}^{2}+{\left(\left(\max[20\log(\frac{\left|AF(\theta_{k})\right|}{\max(AF(\theta_{k}))})]-bb2\right)/bb2\right)}^{2}\right]$$

*where* $\theta_{i}\in [0^{\circ },80^{\circ }]\cup [100^{\circ },180^{\circ }]$ *that is the range of sidelobes in a linear array,* $\theta_{k}$ *is the angle of the deep null and*  $\theta_{k}=104^{\circ }$. bb1 *represents the ideal maximum SLL that is desired for optimization, and bb2 represents the desired ideal sidelobe level at the deep null point, then* $AF(\theta_{k})$ *represents the sidelobe level at*  $k^{\circ }$*. In Equation (19), bb1 = −29 dB, bb2 = −120 dB.*

Table 7 presents a comparative analysis of CFMINFO’s results against those obtained from HAS [33], GA [5], PSO [7], and FPA [30], highlighting SLL and null depth performance. Comparison results indicate that CFMINFO outperforms the referenced arrays in minimizing SLL, achieving a null depth of −125.1 dB at 104°. The AF < −60 dB is considered a null.

**Figure 17:**
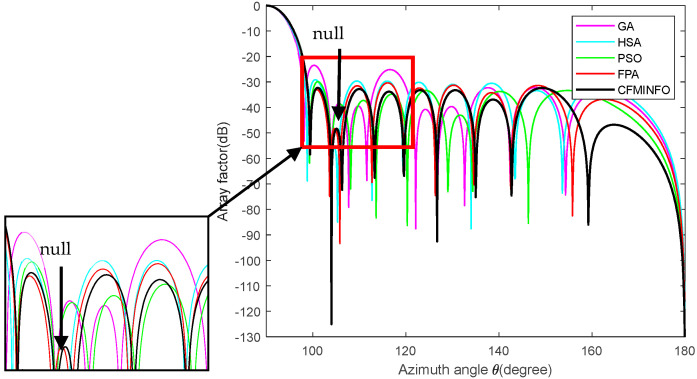
The array factor of a 20-element array with deep nulls optimized by CFMINFO compared to other algorithms.

Figure 17 presents the optimized array patterns at 104° for CFMINFO, GA [5], FPA [30], PSO [7], and HAS [33], illustrating their effectiveness in achieving the target null depth while minimizing SLL.

Table 7 and Figure 17 demonstrate CFMINFO’s superior performance, achieving a maximum SLL of −32.30, which is 1.95–8.84 dB lower than FPA, HAS, PSO, and GA. At the 104° deep null, its SLL is 2.6–27.2 dB lower than that of these algorithms. This finding demonstrates CFMINFO’s superior SLL reduction, highlighting its value in linear array antenna design and application. Table 8 presents the optimized excitation amplitudes from Example 5.

In summary, the CFMINFO algorithm consistently achieves lower maximum SLL with robustness and reliability, demonstrating superior optimization performance over comparable methods. Its precise control and efficient strategy make it a strong candidate for complex antenna array optimization.

## 5. Conclusions

This study presents CFMINFO, an enhanced INFO algorithm with multiple optimization strategies for linear arrays. CFMINFO effectively synthesizes linear array patterns and regulates deep nulls, consistently outperforming GOA, WOA, FATA, CSO, ALO, and other algorithms in SLL reduction, convergence speed, and stability. Moreover, it achieves precise deep nulls in specific antenna directions by optimizing the SLL at the null while controlling the maximum SLL, outperforming IWO. It updates the vector positions via three core mechanisms, integrating Good-Lattice-Point Set initialization, STC chaotic parameterization, and Normal Cloud Mutation strategies. It is gradient-free, converges rapidly, and effectively mitigates local optima traps. Compared to other algorithms, CFMINFO-based pattern synthesis and deep null control optimization deliver superior results while reducing the computational costs in antenna optimization.

In summary, the proposed CFMINFO algorithm exhibits marked superiority in constrained linear array synthesis, outperforming conventional approaches under identical computational budgets. However, several avenues merit future exploration. Integrating deep or reinforcement learning into the CFMINFO framework could enhance adaptive parameter tuning and strengthen global search capability. Methodologically, the approach can be extended from one-dimensional linear arrays to planar and conformal arrays, demonstrating its scalability for higher-dimensional synthesis. Additionally, exploring richer constraint sets, including multi-objective formulations that jointly optimize sidelobe suppression, beamwidth, and pattern robustness, is promising. Finally, from an engineering perspective, assessing the feasibility of real-time optimization and hardware acceleration on FPGA or GPU platforms could facilitate practical deployment in antenna design.

Practical implications:

- Enables stronger interference suppression and precise null steering for linear/sensor arrays with fewer tuning knobs and shorter design cycles.
- Provides a general, gradient-free optimizer readily embeddable in sensor/antenna co-design and other constrained engineering problems.

## Figures and Tables

**Table 1:** Comparison and statistical analysis of test functions.

		CFMINFO	INFO	GWO	DE	SSA	EnMODE
RC15	Mean SD	2994.424 0	2994.471 9.09 × 10^−13^	3005.406 31.28869	2994.471 9.28 × 10^−13^	3035.138 32.99173	2994.424 4.65 × 10^−13^
RC17	Mean SD	0.01267 6.43 × 10^−6^	0.01269 2.34 × 10^−5^	0.01271 1.25 × 10^−5^	0.01278 6.41 × 10^−5^	0.01292 0.000118	0.01271 0
RC19	Mean SD	1.6702 1.87 × 10^−16^	1.6928 1.85 × 10^−9^	1.6951 0.001413	1.7816 0.055348	1.7818 0.078427	1.6707 0.001044
RC20	Mean SD	263.8958 3.36 × 10^−11^	265.1486 0	263.8979 0.001423	263.8959 4.92 × 10^−5^	263.8967 0.001174	263.8958 0
RC23	Mean SD	16.9268 0.130817	17.4067 0.494939	1.19 × 10^93^ 9.71 × 10^92^	5.27 × 10^91^ 4.33 × 10^91^	5.99 × 10^84^ 5.25 × 10^84^	15.5287 1.4617
RC28	Mean SD	14,614.14 9.28 × 10^−12^	14,614.14 9.28 × 10^−12^	14,641.97 18.82234	14,614.14 9.16 × 10^−12^	155,047.3 2292.9683	16,958.2 0
RC31	Mean SD	0 0	5.07 × 10^−10^ 9.32 × 10^−10^	1.1 × 10^−10^ 2.7 × 10^−10^	3.99 × 10^−10^ 4.48 × 10^−10^	2.2 × 10^−9^ 5.34 × 10^−9^	1.05 × 10^−16^ 2.32 × 10^−16^

**Table 2:** The Friedman test average ranking.

Function	DE	GWO	SSA	INFO	CFMINFO	EnMODE
RC15-median	2994.471066	3005.405731	3035.138492	2994.471066	2994.424466	2994.425
RC17-median	0.012784288	0.012717372	0.012923158	0.01269048	0.012667663	0.012719
RC19-median	1.781618882	1.70 × 10^0^	1.78 × 10^0^	1.69 × 10^0^	1.67 × 10^0^	1.670271
RC20-median	263.895917	263.8979391	263.8967495	265.1485657	263.8958434	263.8958
RC23-median	5.27441 × 10^91^	1.19462 × 10^93^	5.9869 × 10^84^	17.40556218	16.9286316	16.11648
RC28-median	14,614.13572	14,641.97128	15,047.30319	14,614.13572	14,614.13572	16,958.2
RC31-median	3.99121 × 10^−10^	1.10099 × 10^−10^	2.20125 × 10^−9^	5.07383 × 10^−10^	0	5.00 × 10^−19^
Friedamn	4.07	4.29	5.14	3.5	1.36	2.64
Rank	4	5	6	3	1	2

**Table 3:** The statistical sidelobe level of position optimization designs for 12 and 32 element linear arrays, utilizing CFMINFO alongside various other algorithms.

	12-Element					32-Element				
	CFMINFO	GOA	WOA	PSO	Conv	CFMINFO	CSO	PSO	FATA	Conv
Best SLL (dB)	−20.06	−19.99	−18.45	−19.71	−13.06	−24.72	−18.20	−18.60	−14.10	−13.50
Worst SLL (dB)	−17.13	−13.63	−16.72	−16.71	\	−8.52	−6.86	−7.14	−5.64	\
Mean SLL (dB)	−19.63	−19.52	−19.24	−19.48	\	−14.72	−14.01	−14.89	−10.69	\
Std. Dev. SLL (dB)	0.59	1.99	0.81	0.69	\	3.77	3.85	4.27	2.17	\

**Table 4:** The position values of the linear arrays with 12 and 32 elements after optimization using CFMINFO and the other algorithms.

		$[x_{1},x_{2},\ldots ,\;x_{N}$] in *λ*
12-element	CFMINFO	[0.2500, 0.5003, 0.9941, 1.3999, 2.0300, 2.7500]
	GOA	[0.2500, 0.5241, 1.0215, 1.4375, 2.0615, 2.7500]
	WOA	[0.2500, 0.5642, 1.0543, 1.3843, 1.9988, 2.7500]
	PSO	[0.2500, 0.5157, 1.0138, 1.4101, 2.0424, 2.7500]
	Conv	[0.2500, 0.7500, 1.2500, 1.7500, 2.2500, 2.7500]
32-element	CFMINFO	[0.304, 0.456, 1.038, 1.320, 1.951, 2.082, 2.658, 3.072, 3.600, 4.028, 4.675, 5.147, 5.877, 6.675, 7.648, 8.486]
	FATA	[0.250, 0.474, 0.652, 1.195, 1.321, 1.746, 2.025, 2.473, 2.531, 2.927, 3.432, 4.124, 4.254, 5.032, 8.348, 8.670]
	PSO	[0.265, 0.685, 1.175, 1.555, 1.985, 2.330, 2.665, 3.055, 3.430, 3.900, 4.380, 4.950, 5.550, 6.240, 7.050, 7.755]
	CSO	[0.288, 0.683, 1.193, 1.520, 1.977, 2.325, 2.689, 3.136, 3.485, 3.954, 4.382, 4.925, 5.482, 6.209, 7.041, 7.750]
	Conv	[0.250, 0.750, 1.250, 1.750, 2.250, 2.750, 3.250, 3.750, 4.250, 4.750, 5.250, 5.750, 6.250, 6.750, 7.250, 7.750]

**Table 5:** The max SLL and excitation amplitudes for both the 10-element array and the 32-element array.

		Maximum SLL (dB)	Optimized Excitation Amplitudes
10-element	CFMINFO	−28.44	[1.0000, 0.8845, 0.6848, 0.4521, 0.2918]
	FPA	−25.33	[1.0000, 0.8979, 0.7178, 0.5002, 0.3833]
	ALO	−26.08	[1.0000, 0.8959, 0.6957, 0.4935, 0.2966]
	PSO	−24.62	[1.0000, 0.9010, 0.7255, 0.5120, 0.4088]
	Conv	−12.90	[1.0000, 1.0000, 1.0000, 1.0000, 1.0000]
16-elemnet	CFMINFO	−29.75	[1.000, 0.957, 0.865, 0.739, 0.605, 0.463, 0.317, 0.288]
	GOA	−28.10	[1.000, 0.958, 0.874, 0.756, 0.627, 0.485, 0.367, 0.349]
	IWO	−26.39	[1.000, 0.976, 0.931, 0.793, 0.660, 0.644, 0.400, 0.409]
	FA	−25.34	[1.000, 0.907, 0.880, 0.753, 0.596, 0.500, 0.366, 0.397]
	Conv	−17.49	[1.000, 1.000, 1.000, 1.000, 1.000, 1.000, 1.000, 1.000]

**Table 6:** Performance comparison of various algorithms for 10-element and 16-element arrays with CFMINFO after 20 runs.

	10-Element					16-Element				
	CFMINFO	FPA	PSO	ALO	Conv	CFMINFO	GOA	IWO	FA	Conv
Best SLL (dB)	−28.06	−25.33	−24.62	−26.08	−12.9	−29.75	−28.10	−26.39	−25.34	−17.49
Worst SLL (dB)	−27.21	−25.30	−20.71	−19.76	\	−28.89	−27.67	−25.35	−24.26	\
Mean SLL (dB)	−27.65	−25.31	−22.69	−22.36	\	−29.07	−27.94	−26.41	−24.61	\
SD SLL (dB)	0.56	0.06	1.86	2.76	\	0.13	0.13	0.05	0.31	\

**Table 7:** A comparative analysis of CFMINFO’s results with those obtained from HSA, GA, PSO, and FPA algorithms.

	CFMINFO	FPA	HSA	PSO	GA
The max SLL (dB)	−32.30	−30.35	−29.14	−29.89	−23.46
Null (dB) (104°)	−125.1	−122.5	−120.9	−103.3	−97.9

**Table 8:** Optimized excitation amplitudes in Example 5.

	Optimized Excitation Amplitudes
CFMINFO	[1.0000, 0.9571, 0.9388, 0.8139, 0.7248, 0.5660, 0.4466, 0.2896, 0.2302, 0.1883]
FPA	[1.0000, 0.9472, 0.9230, 0.8239, 0.7287, 0.5760, 0.4414, 0.2973, 0.2304, 0.2304]
PSO	[1.0000, 0.9918, 0.9123, 0.8040, 0.7475, 0.5617, 0.4698, 0.2828, 0.2536, 0.1296]
HSA	[1.0000, 0.9962, 0.9412, 0.8872, 0.7711, 0.6353, 0.4828, 0.3342, 0.2586, 0.2824]
GA	[1.0000, 0.9132, 0.8452, 0.8610, 0.8475, 0.6589, 0.4986, 0.4050, 0.2826, 0.2476]

## Data Availability

Chen, Zhuo (2025), “CEC2020 Real-World Constrained Engineering Optimization Seven-Problem Suite”, Mendeley Data, V1, https://doi.org/10.17632/nvjkfdycpw.1. Yan Liu (2025). Chaos Fusion Mutation-Based INFO Algorithm for Linear Antenna Array Optimization. IEEE Dataport. https://doi.org/10.21227/1jbz-wt55. https://data.mendeley.com/datasets/nvjkfdycpw/1 (accessed on 3 September 2025).

## Appendix A

1. General linear-array factor (far-field superposition)

Consider a linear arrangement along the x-axis with wavenumber k=2π/λ. The position of the n-th element is $x_{n}\in R$, and its complex excitation is $a_{n}=A_{n}e^{j\alpha_{n}}$, With a progressive scan phase $\beta_{n}$ (for beam steering), the far-field array factor (neglecting the element pattern) is obtained by linear superposition of each element’s contribution with its geometric phase $kx_{n}\cos\theta$:

(A1) $$AF(\theta )=\sum_{n-1}^{N}a_{n}e^{j(kx_{n}\cos\theta +\beta_{n})}=\sum_{n-1}^{N}A_{n}e^{j\alpha_{n}}e^{j(kx_{n}\cos\theta +\beta_{n})}$$

A common choice is $\beta_{n}=(n-1)\beta$ (uniform progressive phase). Equation (A.1) is the general working expression from which the symmetric forms are derived.

2. Symmetric Linear Array with 2N Elements

Consider a symmetric linear array centered at the origin with 2N isotropic elements. Let the n-th symmetric pair be located at $\pm d_{n}$ (decision variables if spacing is optimized), with complex excitations $a_{\pm n}=A_{n}e^{\pm j\alpha_{n}}$. Pairing the conjugate phase terms in the general superposition yields the cosine form

(A2) $$\begin{array}{l} AF(\theta )=\sum_{n-1}^{N}[A_{n}e^{j\alpha_{n}}e^{j(kd_{n}\cos\theta +\beta_{n})}+\sum_{n-1}^{N}[A_{n}e^{-j\alpha_{n}}e^{-j(kd_{n}\cos\theta -\beta_{n})}]\\ =2\sum_{n=1}^{N}A_{n}\cos(\alpha_{n}+\beta_{n}+kd_{n}\cos\theta ) \end{array}$$

where k=2π/λ is the wavenumber, $\beta_{n}$ is the progressive phase for each element; when beam scanning is not required, set $\beta_{n}=0$, and $d_{n}>0$ is the distance of the *n-th* pair from the array center.
